# Encapsulated Microparticles of (1→6)-β-d-Glucan Containing Extract of *Baccharis dracunculifolia*: Production and Characterization

**DOI:** 10.3390/molecules24112099

**Published:** 2019-06-03

**Authors:** Genice Iurckevicz, Débora Dahmer, Vidiany A. Q. Santos, Vaclav Vetvicka, Aneli M. Barbosa-Dekker, Robert F. H. Dekker, Carlos Ricardo Maneck Malfatti, Mário A. A. da Cunha

**Affiliations:** 1 Chemistry Department, Universidade Estadual do Centro Oeste, Rua Simeão Varela de Sá, 03, Vila Carli, CEP, Guarapuava, PR 85040-080, Brazil; genice.iur@gmail.com (G.I.); crmalfatti@gmail.com (C.R.M.M.); 2 Chemistry Department, Universidade Tecnológica Federal do Paraná, Via do Conhecimento, Km 1, CEP, Pato Branco, PR 85503-390, Brazil; debora-dahmer@hotmail.com (D.D.); vidianyqueiroz@yahoo.com.br (V.A.Q.S.); 3 Department of Pathology, University of Louisville, 511 S. Floyd St, Louisville, KY 40292, USA; v0vetv01@louisville.edu; 4 Chemistry Department, CCE, Universidade Estadual de Londrina, CEP, Londrina, PR 86057-970, Brazil; anelibarbosa@gmail.com; 5 Programa de Pós-Graduação em Engenharia Ambiental, Universidade Tecnológica Federal do Paraná, Câmpus Londrina, CEP, Londrina, PR 86036-370, Brazil; xylanase@gmail.com

**Keywords:** bioactivity, encapsulation, experimental design, natural products

## Abstract

β-Glucans are biomacromolecules well known, among other biological activities, for their immunomodulatory potential. Similarly, extracts of *Baccharis dracunculifolia* also possess biological properties and are used in folk medicine for the treatment of inflammation, ulcers, and hepatic diseases. Microparticles containing (1→6)-β-d-glucan (lasiodiplodan) and *B. dracunculifolia* extract were produced and characterized. A 2^3^ factorial design was employed to define the conditions of production of microparticles by atomization. Lasiodiplodan associated with maltodextrin and gum arabic was studied as a matrix material. Microparticles of 0.4 μm mean size and high phenolics content (3157.9 μg GAE/g) were obtained under the optimized conditions. The microparticle size ranged from 0.23 to 1.21 µm, and the mathematical model that best represented the release kinetics of the extract was the Korsmeyer-Peppas model. Diffusional exponent (n) values of 0.64 at pH 7.7 and 1.15 at pH 2.61 were found, indicating particles with a non-Fickian or anomalous transport system, and Super Case II transport, respectively. Thermal analysis indicated that the microparticles demonstrated high thermal stability. The X-ray diffraction analyses revealed an amorphous structure, and HPLC-DAD analysis showed microparticles rich in phenolic compounds: caffeic acid, p-coumaric acid, and catechin. The microparticles obtained comprise a new biomaterial with biological potential for applications in different fields.

## 1. Introduction

Medicinal plants are part of the history of human evolution. More than 50% of all drugs used in modern medicinal treatments are composed of natural products and derivatives thereof [1]. The physicochemical stability is a determining factor in the quality of plant extracts and the transformation of these into dry-powdered form is the most desirable strategy, considering that this form improves its stability and facilitates the manipulation of the material [2]. Techniques for the incorporation of plant extracts within polymer matrices have indicated a good alternative for the improvement of the functionality of medicinal plant extracts [3]. The spray-drying process that involves the dispersion of material inside a coated material is a technique that has been widely used in recent years for the incorporation of extracts into polymer matrices [4].

*Baccharis dracunculifolia*, popularly known in Brazil as alecrim-do-campo (field rosemary) or vassourinha do campo, is a medicinal plant used to treat human disease conditions and from which green propolis is produced by honeybees [5,6,7]. Several biological properties related to *B. dracunculifolia*, including antidiabetic [8], anti-inflammatory [7], bactericidal [9], and antioxidant [10] activities, have been reported in the scientific literature.

The combination of natural products can make it possible to obtain biomaterials with new potential biological functions. In this sense, the association of plant extracts with microbial β-glucans may offer a promising strategy for obtaining new drugs. β-Glucans are carbohydrate biopolymers constituted of repeat d-glucose monomers linked by specific β-glycosidic bonds [11]. These macromolecules have attracted attention from the pharmaceutical industry for their biological functions that can include immunomodulatory, antimutagenic, anticarcinogenic, hypoglycemic, and hypocholesterolemic properties [12]. More recently, d-glucans have been studied as a support, adjuvant or in combination with other materials in drug delivery systems [13]. It is important to note that modified-release drug delivery systems can improve drug efficacy and contribute to the increased bioavailability, solubility, pharmacological activity, stability, and protection against chemical degradation [3,14]. 

Spray-drying is a technique that has been widely used in the development of modified release systems [15,16]. It is a rapid process that does not involve severe heat treatment; therefore, it is a suitable method to preserve biological products, including temperature sensitive products, without their degradation; it also allows for storage at room temperature [17,18]. It is an instantaneous process where spherical and uniform samples can be obtained, and the process can be easily scaled up. Thermal degradation, nutrient loss, and denaturation are minimal, allowing maximum retention of the active principles. Spray-drying is a technique more economical than lyophilization (four to seven times less costly); it also allows coating, complex coacervation, and drying of two different feed solutions during the process [19].

Considering that medicinal plant extracts have been little studied in modified-release systems and that the combination of these with β-glucans could promote obtaining biomaterials with new biological functions, the present study was aimed towards the production of microparticles containing extracts of *B. dracunculifolia* and the fungal exopolysaccharide β-glucan (lasiodiplodan) from *Lasiodiplodia theobromae* matrix metalloproteinase inhibitors (MMPI).

The study included the development and characterization of the microparticles obtained by atomization application using the spray-drying process, and the evaluation of the antioxidant potential and of the kinetics of the release of *B. dracunculifolia* extract from the encapsulated microparticles.

## 2. Results and Discussion 

### 2.1. Encapsulation Yield

**Figure 1:**
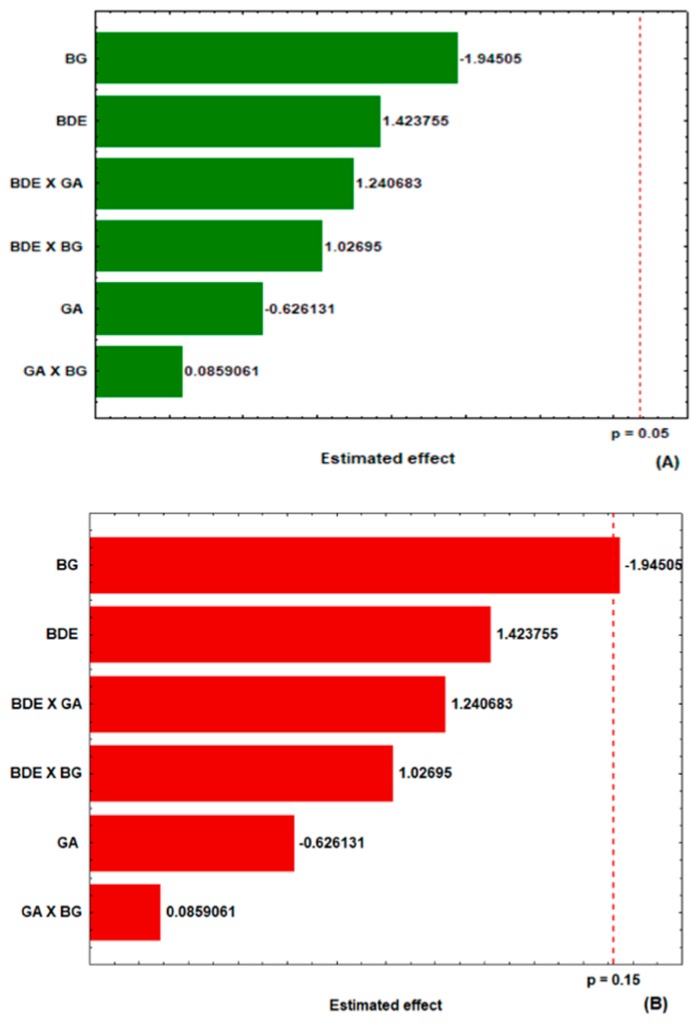
Estimated effects of the concentrations of β-glucan (BG), gum arabic (GA), and *B. dracunculifolia* extract (BDE) on the encapsulation yield of the microparticles at the 95% (**A**) and 85% (**B**) confidence levels.

The effects of the studied variables in the experimental design on the encapsulation yield were plotted in Pareto charts as shown in Figure 1. At a confidence level of 95% (*p* < 0.15) none of the factors examined had a significant effect on the yield of microparticles produced (Figure 1A). However, considering an 85% confidence level (*p* < 0.15), the concentration of (1→6)-β-d-glucan had a significant negative effect on yield (Figure 1B). Within this confidence interval, the increase in β-glucan concentration in the encapsulation mixture did not contribute to the yield of the microparticle production. This phenomenon might be explained by increased viscosity of the encapsulation system as a function of the increased concentration of lasiodiplodan. In the microencapsulation process by spray-drying, the viscosity of the system should be low enough to prevent air inclusion in the particles [20,21].

### 2.2. Total Phenolic Content in the Microparticles

**Figure 2:**
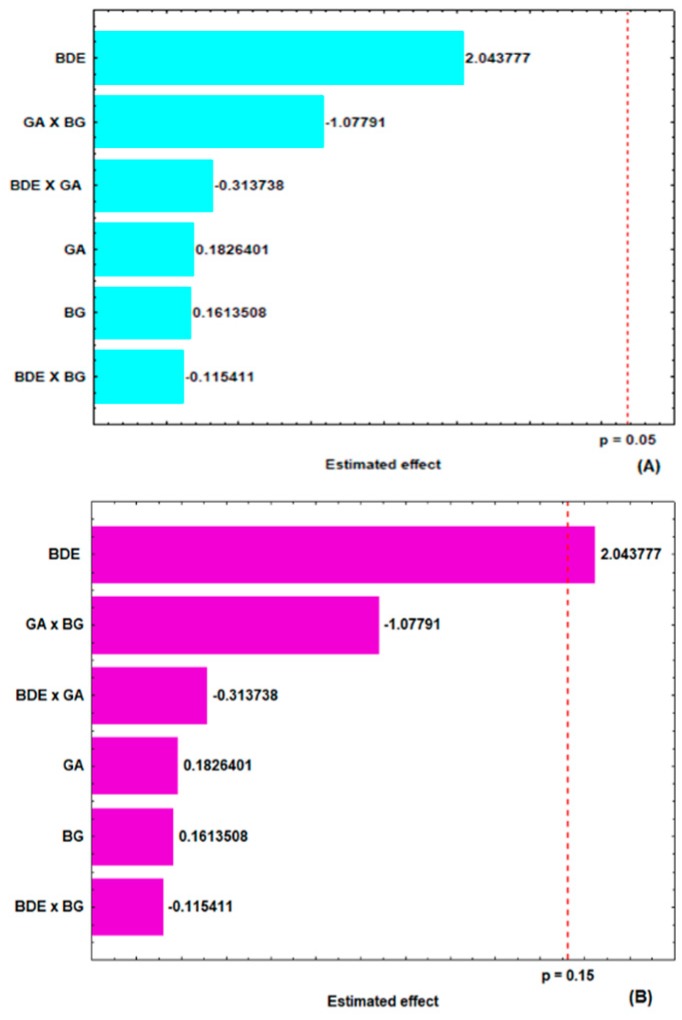
Estimated effects of the concentrations of β-glucan (BG), gum arabic (GA) and *B. dracunculifolia* extract (BDE) on the total phenolic content of the microparticles at the 95% (**A**) and 85% (**B**) confidence levels.

As was observed in the encapsulation yield at a 95% confidence level (*p* < 0.05), no factor had a significant effect on the total phenolic content (Figure 2A) of the microparticles. However, on decreasing the statistical confidence interval to 85% (*p* < 0.15), the variable concentration of *B. dracunculifolia* extract (BDE) showed a statistically significant positive effect (Figure 2B).

In the experimental runs where higher concentrations of BDE (5%) were used, microparticles with higher concentrations of total phenolics were produced (Table 1). In fact, plant extracts contain high concentrations of phenolic compounds, which are secondary plant metabolites [22]. Similar contents of total phenolics were found in run 2 (3154.90 µg GAE/g) and run 6 (3157.90 µg GAE/g) (Table 1). The encapsulation conditions employed in these factorial design experiments were similar, differing only in relation to the β-glucan (lasiodiplodan) content employed. In run 2, 2% of β-glucan was used, while in run 6, a concentration of 5% was used.

Considering the objective of obtaining a biomaterial rich in bioactive compounds, the conditions used in experimental run 6 were assumed to be the best condition for the production of microparticles incorporating the extract of *B. dracunculifolia* and (1→6)-β-d-glucan. It is noteworthy that higher concentrations of β-glucan (1.5%) were employed in this condition, which is interesting considering the biological and functional properties of glucans. In addition, lower cost microparticles could be produced without the incorporation of gum arabic.

### 2.3. Antioxidant Activity

The antioxidant activity found in the microparticles sample obtained from run 6 is shown in Table 2. The microparticles showed scavenging potential of both the DPPH (33.6 μmol Trolox/g) and the ABTS cation radicals (24 μmol Trolox/g), and appreciable antioxidant potential for ferric ion reduction (FRAP: 212.2 μmol FeSO_4_·7H_2_O/g).

### 2.4. Particle Size and Zeta Potential

**Figure 3:**
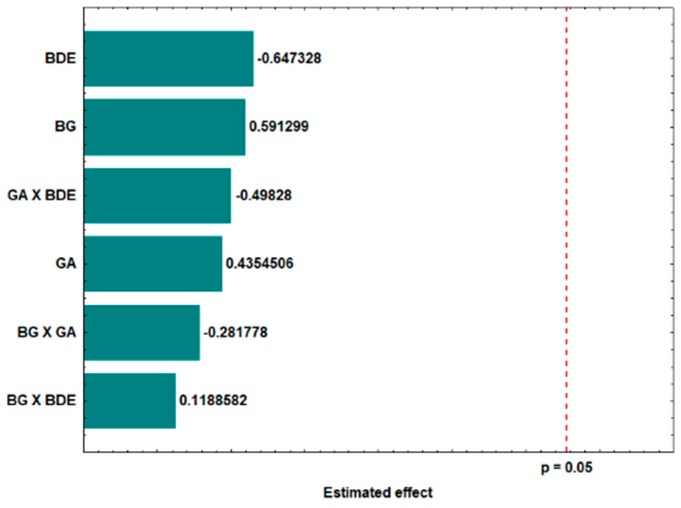
Estimated effects of the concentrations of β-glucan (BG), gum arabic (GA), and *B. dracunculifolia* extract (BDE) on the size distribution of the microparticles formed at the 95% confidence level (*p* = 0.05).

Table 1 shows the average size, polydispersity index and zeta potential of the microparticles formed through the experimental statistical design. None of the independent variables studied showed significant effects on microparticle size (Figure 3). The microparticles ranged in size from 0.24 µm (run 2 sample) to 1.21 µm (run 9 sample); the run 6 sample was obtained using the optimal spray-drying conditions (assumed to be the best) and showed a particle mean size of 0.4 µm.

Larger dimensions (mean diameter range, 7.0–11.5 μm) were reported by Carvalho et al. [23] in microparticles containing jussara (*Euterpe edulis*) extract, when using gum arabic and maltodextrin as matrix materials. Similarly, Krishnaiah et al. [24] obtained microparticles of *Morinda citrifolia* Linn extract encapsulated in maltodextrin and carrageenan with mean diameters (range, 2–5 μm) greater than that found in our study. Li et al. [17] studied five matrix materials (maltodextrin, gum arabic, polyvinyl alcohol, whey protein, and modified starch) using spray-drying technology for lipid encapsulation. Particles with mean sizes that ranged from 1 μm to 350 nm were obtained [17].

The polydispersity index (PdI) represents the varied particle size distribution, and can range from zero (0) in uniform particles to 1.0 in polydisperse particles [25]. In our study, we found a rather large distribution of particle sizes (0.5–1.0). This is possibly related to the high viscosity of the mixture subjected to spray-drying, which also influenced the production yield of the microparticles. In fact, the composition of the matrix material may interfere in the production yield of the microparticles as well as the polydispersity index. In addition, the nature of the matrix material, the concentration of the spray-dried solution, and the location of collection on the spray-drying equipment can influence the size of the microparticles [17].

**Figure 4:**
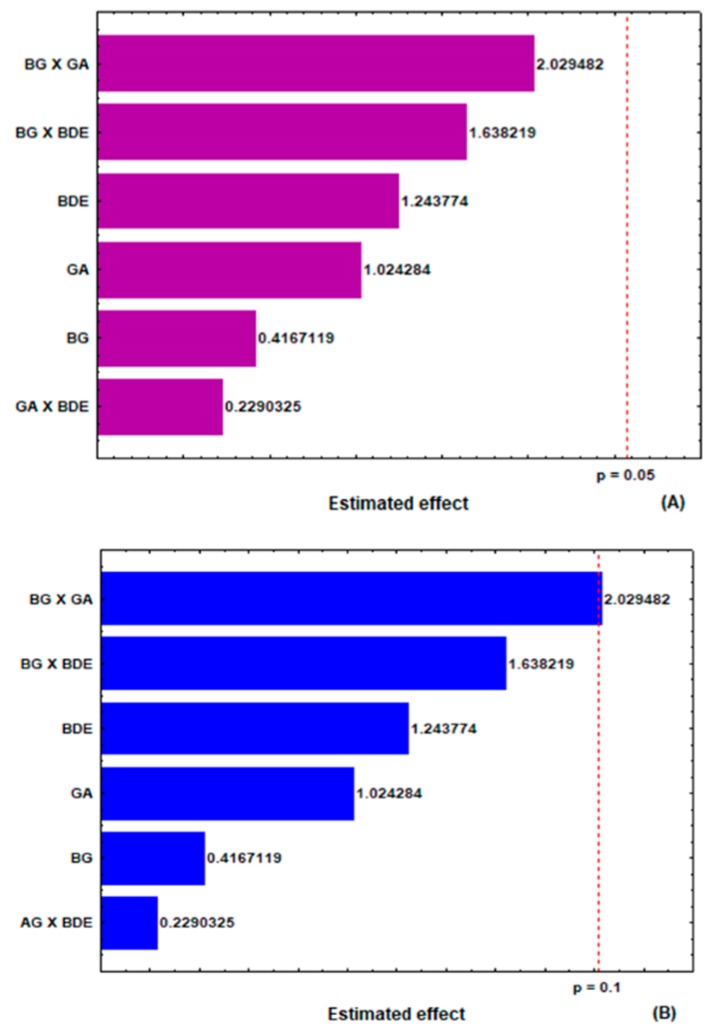
Estimated effects of the concentrations of β-glucan (BG), gum arabic (GA) and *B. dracunculifolia* extract (BDE) on the microparticles zeta potential at the 95% (**A**) and 90% (**B**) confidence levels.

Zeta potential (ZP) is a physicochemical parameter that reflects the electrical potential of particles and provides information about their physical stability. ZP is influenced by the dispersion medium and composition of the particle, and when above ± 30 mV, the surface charges avoid microparticle aggregation and indicate a stable suspension [26]. From the results shown in Table 1 and Figure 4A,B, no factor had a significant effect of first (main effect) or second (interaction of two factors) order on this response at the 95% confidence level (*p* < 0.05). On the other hand, when analyzing the interaction effects between the concentrations of β-glucan and gum arabic at the 90% confidence level (*p* < 0.10), a positive effect on the ZP was observed (Figure 4B). In this context, higher concentrations of β-glucan and gum arabic could contribute to obtaining microparticles with higher ZP.

The microparticles produced presented a ZP that ranged from −15.7 (central point) to −25 mV (run 8; Table 1). In experimental run 6, where the highest concentrations of both β-glucan and BDE were employed, a ZP value of −18.53 mV was observed. Gomes et al. [27] reported on maltodextrin and gum arabic particles coated with egg yolk and a bilayer composed of α-phosphatidylcholine and stearylamine, and found a ZP of 56 mV. However, when only gum arabic and maltodextrin were employed as encapsulating agents, a negative ZP resulted (−16 mV). According to these authors, the negative charge on the surface of those microparticles was attributed to the carboxyl groups of glucuronic acid present in gum arabic, which at neutral pH are deprotonated in ionized form [27].

### 2.5. Thermal and X-ray Diffraction Analyses

**Figure 5:**
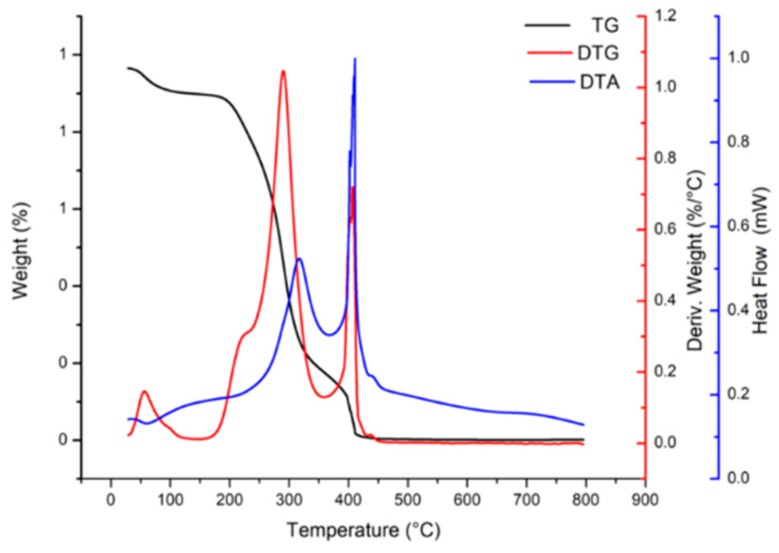
Thermogravimetric (TG) and derived thermogravimetric (DTG) profiles of microparticles.

Figure 5 shows the profiles of thermogravimetry and derived thermogravimetry of the microparticles.

The first thermal event occurred between 28 °C and 124 °C with 2.3% mass loss, which is related to free water loss [28,29]. Such water loss is evidenced through an endothermic peak at 59 °C in the differential thermogravimetric curve (DTA). The second and main event occurred between 164.4 °C and 357 °C, with an expressive mass loss (77.6%) and indicated by an exothermic peak at 316 °C (DTA), which can be associated with fusion and degradation of the matrix polymeric material [30]. The third event occurred between 370 °C and 429 °C with 17% mass loss, confirmed by an exothermic peak at 411 °C at differential thermogravimetric curve, and being attributable to the final decomposition of the microparticles [31]. Ballesteros et al. [32] studied the encapsulation of antioxidant phenolic compounds from spent coffee grounds by freeze-drying and spray-drying using maltodextrin, and a mixture of these components as coating materials, and observed similar peaks; an endothermic peak between 25 °C and 180 °C related to water evaporation. A second mass loss event was observed between 190 °C and 370 °C (exothermic peak at around 300 °C), and could be related to depolymerization of the materials. The results of the thermal analysis demonstrated that the microparticles have high thermal stability, considering the usual temperature standards employed in the production processes of pharmaceutical and food industries.

**Figure 6:**
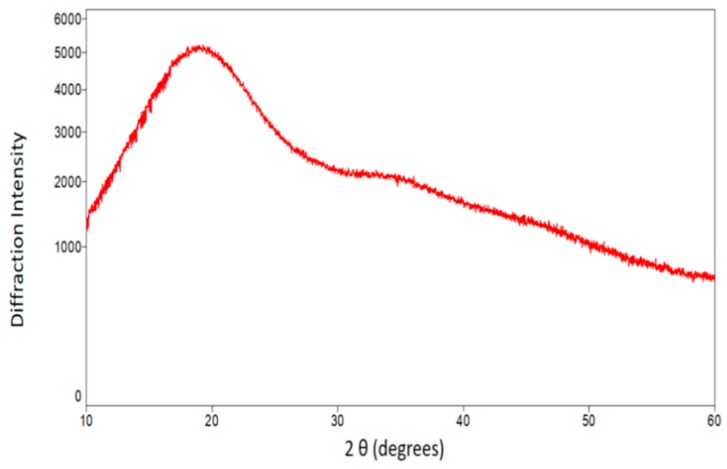
X-ray diffraction patterns of the microparticles.

X-ray diffraction analyses showed that the microparticles have an amorphous structure (Figure 6) indicated by a broad peak observed at approximately 19° (2θ). Similarly, Ballesteros et al. [32] found a low degree of crystallinity in samples of phenolic compounds extracted from spent coffee grounds and encapsulated in maltodextrin and gum arabic. Such samples showed a diffractographic pattern characterized by a very broad peak at approximately 18° (2θ), as also found in the present study. In fact, microparticles with amorphous structure were to be expected, considering the polymeric composition of the matrix materials (lasiodiplodan and gum arabic associated with maltodextrin). It is important to note that amorphous drug systems are widely used to improve the bioavailability and oral and aqueous solubility of a drug [33].

### 2.6. FT-IR Spectroscopy

**Figure 7:**
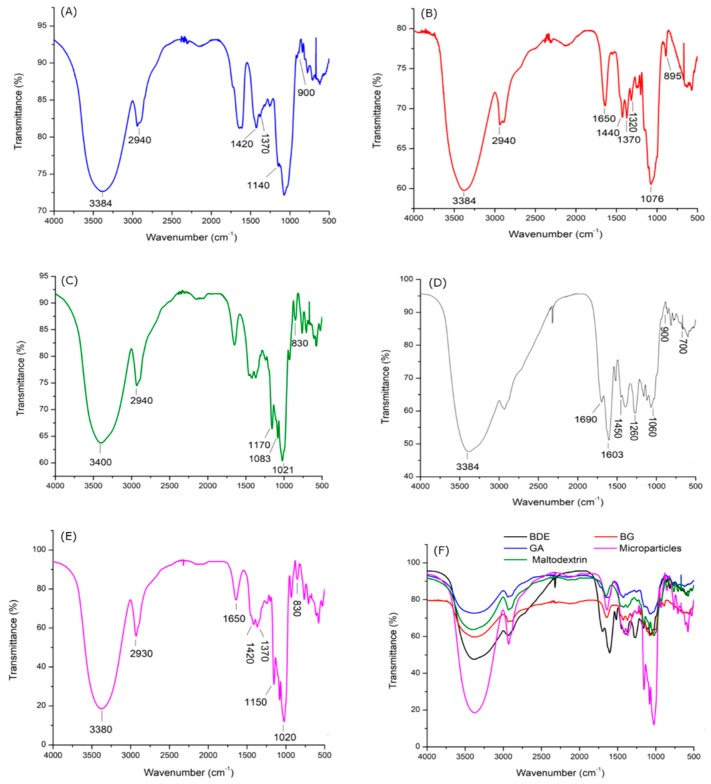
FT-IR spectra of gum arabic (**A**), β-glucan (**B**), maltodextrin (**C**), *B. dracunculifolia* extract (**D**); microparticles obtained in run 6 (**E**), and overlapped spectra (**F**).

Fourier-transform infrared (FT-IR) spectroscopy analysis was used to assess differences and similarities between the microparticles and the component materials constituting their matrices and the lyophilized extract (BDE) (Figure 7).

The FT-IR spectra of maltodextrin, gum arabic and lasiodiplodan (Figure 7) showed bands characteristic of polysaccharides. Strong intensity bands in the region between 3000–3600 cm^−1^ corresponded to –OH stretching vibration [34,35]. Bands in the 2940 cm^−1^ region (Figure 7A–F) were attributed to the C–H stretching vibration [36,37]. The band around 1400–1370 cm^−1^ in gum arabic (Figure 7A) was assigned to the –OH bending and C=O symmetrical stretching of the carboxyl groups present in glucuronic acids, a constituent of the structure of gum arabic. Furthermore, a broad band was observed in the region between 900 cm^−1^ and 1140 cm^−1^ that is characteristic of gum arabic and is due to the C–O stretching of arabinogalactans [38]. It is important to note that gum arabic is a complex macromolecule, which in addition to the polysaccharide constitution also contains small amounts of protein. However, specific signals characteristic of protein groups corresponding to the amino group were not observed in the FT-IR spectra of the gum arabic sample, and this could be due to overlapping signals with the hydroxyl group (–OH) from the polysaccharide [34]. Band at 1320 cm^−1^ corresponding to –OH bending of the C–OH group [39] was verified in the (1→6)-β-d-glucan sample (Figure 7B). The bands next to 1650 cm^−1^ (Figure 7A–E) refer to the glucose ring [31,40], and the bands observed around 1370–1450 cm^−1^ refer to the symmetrical deformation of C–OH and CH_2_ groups [31]. The band observed at 1076 cm^−1^ (Figure 7B) is due to the symmetrical stretching vibrations of C–O–C bonds, characteristic of sugars, and the band at 1243 cm^−1^ is characteristic of asymmetric vibrations [31]. The band observed at 895 cm^−1^ was characteristic of a β-glucan [41].

Maltodextrin (Figure 7C) is a mixture of oligosaccharides composed of a chain of 5 to 10 glucose units per molecule and is derived by starch hydrolysis [42]. Maltodextrin presents bands at 1021 cm^−1^, 1083 cm^−1^, and 1158 cm^−1^, which are related to the C–O stretching and C–OH bending vibrations [43]. The bands observed between 800 cm^−1^ and 1200 cm^−1^ (see Figure 7C) are attributable to the C–O bond stretching, a characteristic of the anhydroglucose ring [42].

The infrared spectrum of *B. dracunculifolia* extract (Figure 7d) was obtained with the purpose of comparison with the spectrum of the microparticles considering their complex composition, which includes flavonoids and phenolic acids [10]. Several absorption bands, including a wide band at 3384 cm^−1^ assigned to –OH stretching vibration from alcohols or phenolic compounds, bands at 1603 and 1450 cm^−1^ corresponding to C=C axial deformation in aromatic compounds, and the band around 1690 cm^−1^ related to conjugated carbonyl or hydrogen bonding [44], were also observed.

According to Silva et al. [44], such bands are generally related to flavonoid compounds. Alcohols and phenols exhibit vibrational stretching with strong bands between 1260 cm^−1^ and 1000 cm^−1^ [45]. In the 900–700 cm^−1^ region, the bands observed are related to CH angular deformations in aromatic groups [45].

### 2.7. Kinetics of the Release of B. dracunculifolia Extract from the Microparticles

**Figure 8:**
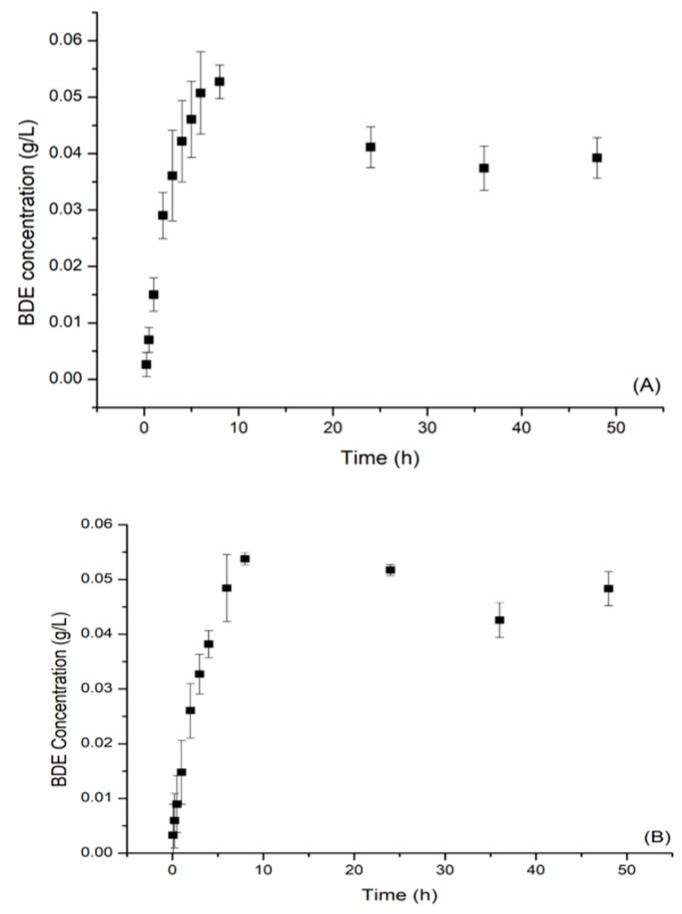
*B. dracunculifolia* extract release kinetic curves at pH 2.6 (**A**) and pH 7.7 (**B**).

The sample produced in run 6 (Table 1), which presented the highest concentrations of total phenolics, was characterized for the release of *B. dracunculifolia* extract, and the release profile into the medium was examined as a function of time and pH (Figure 8). A fast release of BDE was observed to occur at the beginning of the assay profile and was followed by a sustained release; a biphasic release characteristic [46], and appeared to be influenced by pH of the medium (Figure 8A,B). Over the first 10 h, the release of the extract was more pronounced, but thereafter a reduction in the release rate followed by a sustained release occurred.

Several mechanisms can control the release of drugs, such as diffusion, erosion, or osmosis, and several mathematical models have been proposed and can be used to explain the release profile [47]. These models were developed to try to elucidate the swelling and release kinetic processes of the polymer particles, among the models were first order [48], and those proposed by Korsmeyer et al. [49] and by Siepman and Peppas [50], which have been reported in several studies [51,52]. The release kinetic studies provide information for the interpretation of the relationship between the structure of the studied materials and the transport mechanism involved, and it is fundamental to relate transport at the molecular level with the macroscopic information [53].

According to Rigter and Peppas [54], the shape and size distribution of the microparticles significantly influence the release kinetics so that the values for the kinetic constant cannot be limited. The kinetic data were adjusted to mathematical models of first order, Korsmeyer and Peppas, and Higuchi as shown in Table 3.

The models that better represented the release profile of the BDE from the microparticles were those of Korsmeyer and Peppas, and Higuchi, as shown in Table 3, through the (R^2^) correlation coefficients. By the Korsmeyer and Peppas model, which demonstrated the best fit of the data, diffusional exponent (n) values of 0.64 (pH 7.70) and 1.15 (pH 2.61) were found. The diffusional exponent (n) values vary according to the system geometry of the particles, with values of n = 0.43 for a sphere, n = 0.45 for a cylinder, and n = 0.5 for a thin film with Fickian diffusion [55,56]. Diffusional exponent (n) values of 0.89 indicate a Case II transport, and values greater than 0.89 characterize Super Case II transport [57], which is controlled by diffusion, polymer chain relaxation, and erosion [58]. In this model, the “K” constant is considered the release velocity and reflects the geometrical structural characteristics of the system [59]. As observed in Table 3, conditions of acid pH contributed to a release mechanism of the type Super Case II (n = 1.15), which is related to polymer relaxation and erosion. Conversely, under conditions close to neutrality (pH 7.70), a release mechanism of the type non-Fickian or anomalous (n = 0.64) was observed.

### 2.8. Scanning Electron Microscopy—SEM

**Figure 9:**
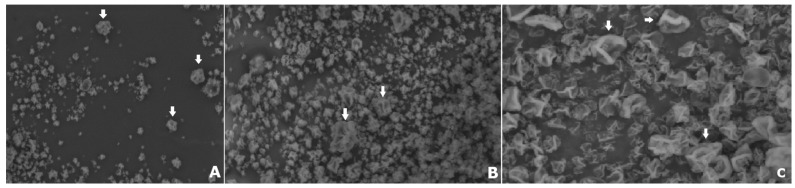
SEM micrographs at magnification of 1500X (**A**,**B**) and 3000X (**C**). Arrows indicating particles similar to raisins.

SEM micrographs revealed particles (Figure 9) with irregular and rough surfaces similar to raisins, and did not present uniform dimensions (diameter). Such features are associated with the possible incorporation of air inside the particles that occur during the atomization process [60]. Commonly, particles obtained by spray-drying present in the form of hollow spheres. The formation of vacuoles is a consequence of the expanding air bubbles trapped within the droplet, and is accompanied by the shrinkage of the coated material on the particle, which occurs as a function of drying the particle [61]. The thermal expansion of air or steam inside the drying particles is associated with the drying rate and the viscoelastic properties of the matrix [62]. Uniform spherical particles are most commonly obtained when more viscoelastic material is employed as the coating matrix [61]. The elasticity of the matrix during the drying process promotes greater homogeneity and uniformity of the particle wall.

### 2.9. Chromatographic Profile of the Encapsulated Phenolic Compounds

**Figure 10:**
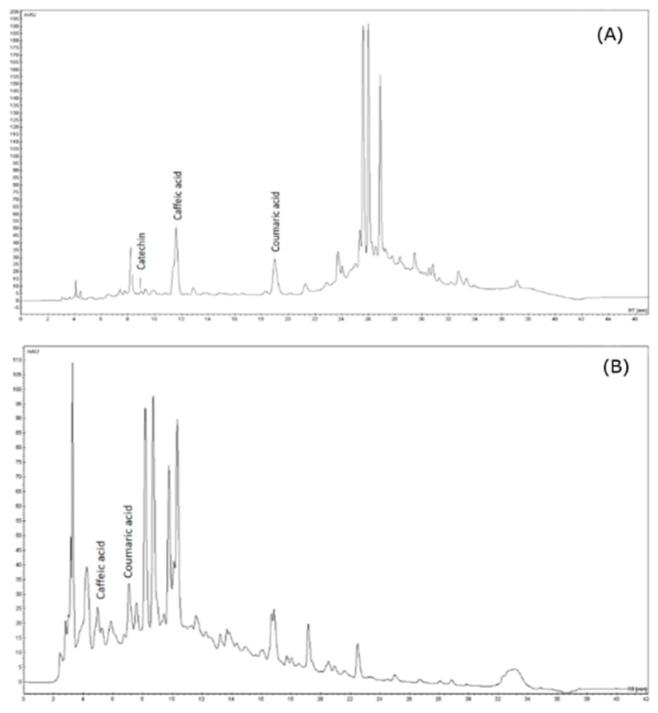
Representative HPLC chromatograms of phenolic compounds extracted from microcapsules and measured in a UV detector at 280 nm (catechin), 300 nm (coumaric acid) and 320 nm (caffeic acid). (**A**) protocol 1 and (**B**) protocol 2.

The microparticles were analyzed by HPLC-DAD after extraction and subjected to two different analysis protocols. Protocol 1 was developed for the detection and analysis of phenolic acids, while protocol 2 detected flavonoids (Figure 10 and Table 4). A major component observed was identified as caffeic acid (17.16 mg/L) followed by p-coumaric acid (16.86 mg/L). These compounds were also identified in the work of Rezende et al. [63] and Sousa et al. [64] in *B. dracunculifolia* extract. Catechin (6.06 mg/L) is commonly found in species of the genus *Baccharis*; e.g., *B. uncinella*, *B. dentata*, and *B. anomala* [65]. The presence of these bioactive compounds identified by HPLC analysis indicate the resistance of the microparticles to the temperature conditions employed in the atomization process during spray-drying.

## 3. Materials and Methods

### 3.1. Botanical Material and Reagents

*B. dracunculifolia* grown on the campus of Universidade Estadual do Centro-Oeste Paraná, at Guarapuava, Paraná Brazil; coordinates: 1.120 m altitude, 50°39′42.39″ West Longitude and 25°32′7.24″ South Latitude), was collected in November 2017. The voucher specimen was deposited in the Herbarium of Universidade Estadual de Ponta Grossa, Ponta Grossa, Paraná Brazil, and registered under the number 18173.

Folin-Ciocalteu reagent, 1,1-diphenyl-2-picrylhydrazyl (DPPH), methanol, potassium persulfate, gallic acid, 6–hydroxyl-2,5,7,8-tetramethylchroman-2-carboxylic acid (Trolox), 2,2′-azino-bis (3-ethylbenzothiazoline-6-sulfonic acid) (ABTS), and gum arabic were obtained from Sigma-Aldrich (St Louis, MO, USA). Maltodextrin (Mor-rex 1910-10 DE, food grade) was kindly provided by Plury Química (Diadema, SP, Brazil). Lasiodiplodan, ((1→6)-β-d-glucan of MW 1.4 × 10^6^ Da) was produced by submerged fermentation on liquid medium containing glucose (20 g/L) as carbon source in (shake flasks), and isolated from the fermentation broth by precipitation with ethanol as described by Cunha et al. [66]. All reagents used in this study were of analytical grade.

### 3.2. Production of B. dracunculifolia Extract

An extract of *B. dracunculifolia* was produced according to the conditions optimized in a previous study. The bioactive compounds present in dehydrated leaves of the plant were ground and extracted with methanol, (1:4 ratio of mass of plant material to volume of 95% methanol) in an orbital incubator (TE-4200, Tecnal, Piracicaba, São Paulo, Brazil) for 20 min at 70 °C. Solvent from the plant extract was removed under vacuum by rotary evaporation (TE-210, Tecnal, Piracicaba, São Paulo, Brazil) at 45 °C. The material extract was frozen at −50 °C, and then lyophilized.

### 3.3. Production of Encapsulated Microparticles by Factorial Design Matrix

Microparticles were produced according to Salgado et al. [67] with adaptations. Gum arabic and lasiodiplodan were separately dissolved in water at 60 °C by stirring (350 rpm) for 20 min and 24 h, respectively. Maltodextrin was dissolved in water at room temperature and stirred for 20 min. Then the solutions (100 mL) of gum arabic and lasiodiplodan solutions were mixed with the maltodextrin solution (200 mL) at high speed in a Ultraturrax homogenizer (Ika, Staufen, Germany) at 20,000 rpm for 20 min [67]. The lyophilized extract of *B. dracunculifolia* was next dispersed in the above solution at 12,000 rpm agitation, and the solution was spray-dried using a Buchi Mini Spray Dryer B-290 (Flawil, Switzerland) employing a feed flow of 83 L/h at a compressed air pressure of 0.55 bar. The drying conditions were set at 130 °C inlet and 87 °C outlet [68]. The microparticles were collected and placed in amber glass bottles for further characterization analyses.

The concentrations of gum arabic, β-glucan (lasiodiplodan), and the lyophilized extract (BDE) varied according to the 2^3^ full factorial design as described in Table 1. Maltodextrin concentration was maintained at 10% (*w*/*v*) in all experimental runs.

### 3.4. Determination of Total Phenolic Content and Antioxidant Activity

The microparticle samples were extracted according to protocol described by Saikia et al. [69] with some modifications. Samples of 500 mg of microparticles were accurately weighed and dispersed in 5 mL of a mixture containing absolute ethanol, glacial acetic acid, and distilled water (50:8:42). The tubes were vortexed for 1 min, and the samples filtered through 0.45 µm Millipore membrane (Merck KGaA, Darmstadt, Germany). The filtrates were used in the analysis of the total phenolics content and antioxidant activity.

#### 3.4.1. Total Phenolics Content in the Microparticles

The total phenolics content was determined by Folin-Ciocalteu method as described by Singleton and Rossi [70]. Volumes of 2.5 mL of Folin-Ciocalteu reagent solution (10%, *v*/*v*), 0.5 mL of microparticle filtrate, and 2 mL of sodium carbonate solution (4% *w*/*v*) were mixed in a test tubes, and kept at room temperature in the dark for 2 h. Absorbance was read at 750 nm in a UV/VIS Digilab—Hitachi U-2800 Spectrophotometer (Lambda Advanced Technology, Wembley, UK). In the blank tube, the filtrate from microparticles was replaced by 0.5 mL of distilled water. Gallic acid was employed as a standard, using a calibration curve within the concentration range of 0–100 mg (R^2^ = 0.9996), and the results were expressed as gallic acid equivalents (µg GAE/g microparticles).

#### 3.4.2. DPPH Free Radical Scavenging Activity

The 1,1-diphenyl-2-picrylhydrazyl (DPPH) scavenging activity of microparticles was evaluated according to Brand-Williams et al. [71]. Aliquots of 0.5 mL of filtrate from the microparticles, 0.3 mL DPPH and 3 mL methanol were homogenized in test tubes, and then kept in the dark at room temperature for 60 min. Methanol was used as the blank. Control sample contained a mixture of 0.3 mL of DPPH radical solution and 3.5 methanol. Trolox was used as the reference antioxidant standard, and the results were expressed in µmol/L Trolox equivalent/g of microparticles calculated from a calibration curve (15–150 µmol Trolox/L, R^2^ = 0.996).

#### 3.4.3. ABTS Free Radical Scavenging Activity

The ABTS cation free radical scavenging capacity was determined according to Huang et al. [72]. ABTS cation radical was generated by mixing 5 mL of ABTS solution (7 mmol/L) and 88 μL of potassium persulfate solution (2.45 mmol/L) in test tubes, which were then left in the dark at room temperature for 16 h. An aliquot of 3 mL of this solution was diluted with methanol until absorbance 0.700 at 734 nm, and then 3 mL this solution was mixed with 30 μL of filtrate from the microparticles followed by homogenization, and the homogenate kept in the dark for 6 min. Methanol was used as a blank. Trolox antioxidant standard (100–2000 µmol/L, R^2^ = 0.998) calibration curve was used to determine the results, expressed as Trolox equivalent µmol/L per g of microcapsules.

#### 3.4.4. Ferric Reducing Antioxidant Power (FRAP) Assay

The ferric reducing antioxidant power (FRAP) was determined according to Wootton-Beard and Ryan [73]. An aliquot filtrate (90 µL) was mixed with 2700 µL of FRAP solution and 270 µL of distilled water. This solution was homogenized in a test tube and kept in the dark for 30 min at 37 °C. FRAP reagent was used as the blank. The results were determined from a calibration curve of ferrous sulfate (5–50 µmol/L, R^2^ = 0.992). Absorbance was measured at 595 nm, and the Fe^+3^ to Fe^+2^ reducing power was expressed as μmol FeSO_4_·7H_2_O per g of microparticles.

### 3.5. Kinetics of the Release of B. dracunculifolia Extract from the Microparticles

The release kinetics of the plant extract from the microparticles was evaluated by measuring the extract content released into the medium (buffer solutions) over the contact time. A sample of 350 mg of microparticles were dispersed in 10 mL of buffer solution pH 2.6 or pH 7.5 (pH 2.6: sodium acetate buffer; pH 7.6: sodium phosphate buffer—PBS). The microparticles dispersed in the buffer solutions were immersed in 150 mL of the respective buffer, and were submitted to dialysis in cellulose membranes (12,000 Da, 1.3 in diam., Sigma-Aldrich).

At predetermined intervals (5 min, 15 min, 30 min, 1 h, 2 h, 3 h, 4 h, 6 h, 8 h, 24 h, 36 h, and 48 h), aliquots of 3 mL were removed from the solution for analysis, and the same volume of dissolution medium was restored to solution to maintain the sink condition to avoid the decrease of the intrinsic dissolution rate caused by the saturation proximity [74]. The mass of the lyophilized extract released was analyzed on a UV/VIS Digilab—Hitachi U-2800 Spectrophotometer (Lambda Advanced Technology) with reading at 315 nm, and correlation with a *B. dracunculifolia* solution calibration curve (0.0975 to 90 mg/L, R^2^ = 0.99). To characterize the release mechanism, the kinetic parameters were determined using the models: first order [48], Korsmeyer-Peppas [49] and Higuchi [50].

### 3.6. HPLC-DAD Analysis

The profile of phenolic compounds released from the microcapsules was analyzed on a Varian 920 LC HPLC system (Varian Inc., Walnut Creek, CA, USA) coupled to photodiode array detector (DAD), and using a reverse-phase C-18 RP (250 × 4.6 mm × 5 μm) column (Microsorb MV-100, Agilent Technologies, Wilmington, DE, USA). The injection volume was 10 μL at a concentration 7.54 mg/mL and the column was kept at 30 °C.

In order to identify a higher number of bioactive compounds, two protocols of analysis were used:

Protocol 1: A gradient mode system was used to elute the samples from the HPLC column at a flow rate of 1 mL/min. Solvent A: acidified water with acetic acid (2%, *v*/*v*). Solvent B: acetonitrile: water: acetic acid at 58:40:2 (*v*/*v*). The solvent gradient mixtures were: mobile phase A 95% and B 5% (2 min); A 80% and B 20% (13 min); A 75% and B 5% (10 min); A 5% and B 85% (7 min); A 5% and B 95% (4 min); A 95% and B 5% (9 min).

Protocol 2: The mobile phase consisted of a mixture of solvents A (water) and B (acidified methanol with *o*-phosphoric acid) using a flow rate of 1 mL/min. The solvent gradient mixtures used were: mobile phase A 70% and B 30% (15 min); A 36% and B 64% (26 min); A 25% and B 75% (28 min); A 5% and B 95% (32 min); and to B 70% and A 30%.

The peak areas were determined by reading the absorbance at 280 nm for the catechin, 300 nm for coumaric acid, and 320 nm for caffeic acid.

### 3.7. Infrared Spectroscopy Analysis (FT-IR)

The microcapsules were ground and mixed with spectroscopic grade potassium bromide. The mixture was pressed (7 Kgf for 7 min) to result in a compact and clear disc, and was analyzed in the FT-IR Spectrophotometer Frontier (Perkin Elmer, Shelton, CT, USA) in the region of 4000–400 cm^−1^, with a resolution of 4 cm^−1^ and 32 accumulated scans.

### 3.8. X-ray Diffraction Analysis

X-ray diffractogram patterns were recorded on a MiniFlex600 diffractometer (Rigaku, Tokyo, Japan) using copper radiation font (CuKα = 1.5418 Å), 30 mA current, 40 kV voltage, scanning range of 10° to 60° (2*θ*), 0.02° step at 2*θ*, and a scanning speed of 0.5°/minute speed.

### 3.9. Scanning Electron Microscopy (SEM)

Micrographs were obtained in a scanning electron microscope (Hitachi TM3000, Irving, TX, USA). The microcapsules were fixed in a holder with carbon tapes, and images were taken at magnifications of 1000× and 1500×.

### 3.10. Thermal Analysis

The microcapsules were submitted to thermogravimetry, differential thermal analysis, and thermogravimetric analysis between 25 °C and 800 °C, in a synthetic air atmosphere, with a flow rate of 50 mL/min and a heating rate of 10 °C/minute in a SDT Q600 thermal analyzer equipment (TA Instruments, New Castle, DE, USA).

### 3.11. Microparticles Size Measurement and Zeta Potential

The particle size distribution and ZP were evaluated in a Zetasizer Nano, ZS90 instrument (Malvern, UK). Samples were diluted in 0.1 mol/L KCl at a concentration of approximately 0.025% (*v*/*v*).

## 4. Conclusions

Microparticles containing (1→6)-β-d-glucan (lasiodiplodan) and *B. dracunculifolia* extract were produced by a spray-drying method. The microparticles presented high phenolic contents and antioxidant potential, when formulated with maltodextrin (10%, *w*/*v*) as a matrix material along with lasiodiplodan (1.5% (*w*/*v*) and a lyophilized extract of *B. dracunculifolia* leaves (5.0%, *w*/*v*). The high antioxidant activity of the microparticles indicate that spray-drying process, even conducted at relatively high temperatures, bore no negative influence either on the total phenolic content or on the antioxidant activity. The microparticles ranged in size from 0.24–1.21 μm; a range considered adequate for the methodology used. The polydispersity index of the microparticles was high, which may be related to the high viscosity of the solution of lasiodiplodan. In this study, the model that best represented the release profile kinetics was the Korsmeyer and Peppas model. The diffusional exponent (n) values in neutral pH dissolution medium of 0.64 displayed a non-Fickian or anomalous transport system, while that of 1.15 indicated a Super Case II transport phenomenon. High-performance liquid chromatography using acidified water and acetonitrile: water: acetic acid as eluents in a gradient system allowed the separation and identification of three phenolic compounds—catechin (6.06 mg/L), caffeic acid (17.16 mg/L), and p-coumaric acid (16.86 mg/L)—in the microparticles. The microparticles obtained constitute a new biomaterial that in addition to containing microbial β-glucan, a known immunomodulatory biomacromolecule, was rich in phenolics compounds and presented high antioxidant potential.

## Figures and Tables

**Table 1:** Experimental arrangement based on a statistical 2^3^ full factorial design.

Runs	Independent Variables			Dependent Variables			
	BG * (%)	GA ^#^ (%)	BDE ^&^ (%)	Total Phenolics	Size	Polydispersity Index	Zeta Potential
				(µg GAE/g)	(μm)	(PdI)	l (mV)
1	0.5 (−1)	0.0 (−1)	2.0 (−1)	762.40 ± 0.0	0.29 ± 0.2	0.57 ± 0.02	19.47 ± 2.5
2	0.5 (−1)	0.0 (−1)	5.0 (1)	3154.90 ± 4.5	0.24 ± 0.1	0.55 ± 0.04	19.83 ± 0.4
3	0.5 (−1)	10 (1)	2.0 (−1)	2556.40 ± 26.9	0.37 ± 0.2	0.79 ± 0.2	18.87 ± 0.9
4	0.5 (−1)	10 (1)	5.0 (1)	3040.00 ± 20.9	0.27 ± 0.2	0.57 ± 0.1	17.27 ± 0.9
5	1.5 (1)	0.0 (−1)	2.0 (−1)	2410.10 ± 11.9	0.38 ± 0.2	0.70 ± 0.07	15.70 ± 1.0
6	1.5 (1)	0.0 (−1)	5.0 (1)	3157.90 ± 22.4	0.40 ± 0.2	0.72 ± 0.07	18.53 ± 2.3
7	1.5 (1)	10 (1)	2.0 (−1)	1277.30 ± 29.0	0.64 ± 0.4	0.79 ± 0.3	18.80 ± 4.0
8	1.5 (1)	10 (1)	5.0 (1)	3098.20 ± 10.4	0.37 ± 0.2	0.75 ± 0.02	25.03 ± 3.2
9	1.0 (0)	5.0 (0)	3.5 (0)	1992.20 ± 6.0	1.2 ± 0.7	1.00 ± 0.0	16.90 ± 2.1
10	1.0 (0)	5.0 (0)	3.5 (0)	1146.00 ± 10.4	0.57 ± 0.3	0.89 ± 0.1	16.57 ± 2.8
11	1.0 (0)	5.0 (0)	3.5 (0)	1499.70 ± 9.0	0.72 ± 0.4	0.97 ± 0.06	15.37 ± 1.9
12	1.0 (0)	5.0 (0)	3.5 (0)	2983.30 ± 3.0	0.88 ± 0.5	0.89 ± 0.2	17.17 ± 0.4

Concentrations of * BG: β-glucan (lasiodiplodan), ^#^ GA: gum arabic, and ^&^ BDE: *B. dracunculifolia extract*.

**Table 2:** The antioxidant activity of the microparticles produced by experimental run 6 as expressed using different methods of measurement.

Method	Antioxidant Activity
DPPH *	33.6 µmol Trolox/g ± 0.7
ABTS ^#^	24 µmol Trolox/g ± 0.2
FRAP ^&^	212.2 µmol FeSO_4_·7H_2_O/g ± 19.0

* DPPH and ^#^ ABTS scavenging activities expressed as µmol Trolox equivalents/g; ^&^ FRAP reducing power expressed as μmol FeSO_4_·7H_2_O/g.

**Table 3:** Models of the release kinetics of *B. dracunculifolia* extract.

Phenolics Release Models	pH of the Medium	
	2.61	7.70
First order	R^2^ = 0.96 K = 0.4959 (h^−1^) y = −0.4959x − 2.7853	R^2^ = 0.96 K = 0.4231 (h^−1^) y = −0.4231x − 2.8863
Korsmeyer-Peppas	R^2^ = 0.99 K = 0.27 n = 1.15 y = 1.1517x − 1.3199	R^2^ = 0.99 K = 0.29 n = 0.64 y = 0.6457x − 1.2211
Higuchi	R^2^ = 0.99 K = 0.4743 y = 0.4743x − 0.169	R^2^ = 0.96 K = 0.15 y = 0.1498x + 0.1161

R^2^: correlation coefficient; K: constant kinetics of *B. dracunculifolia* bioactive release; n: diffusional exponent; x: linear coefficient of the model.

**Table 4:** Chromatographic parameters of the phenolic compounds analyzed by HPLC-DAD.

Standard	RT (min)	ʎ_max_ (nm)	Regression Equation	R^2^ Value	Concentration (mg/L)
Protocol 1					
Catechin	8.96	276	Y = 0.1794x − 0.156	0.9945	6.06
Protocol 2					
Caffeic acid	11.57	320	Y = 1.0138x − 1.229	0.9836	17.16
*p*-Coumaric acid	19.01	309	Y = 1.7784x − 3.2239	0.9812	16.86

Data are presented as mean ± SEM; n = 3; RT: retention time; R^2^: coefficient of determination (R-squared).

## References

[1] Pan S.-Y., Zhou S.-F., Gao S.-H., Yu Z.-L., Zhang S.-F., Tang M.-K., Sun J.-N., Ma D.-L., Han Y.-F., Fong W.-F. (2013). New Perspectives on how to discover drugs from herbal medicines: CAM’s outstanding contribution to modern therapeutics. Evid. Based. Complement. Alternat. Med..

[2] Teixeira C.C.C., de Freitas Cabral T.P., Tacon L.A., Villardi I.L., Lanchote A.D., de Freitas L.A.P. (2017). Solid state stability of polyphenols from a plant extract after fluid bed atmospheric spray-freeze-drying. Powder Technol..

[3] Gallo L., Piña J., Bucalá V., Allemandi D., Ramírez-Rigo M.V. (2013). Development of a modified-release hydrophilic matrix system of a plant extract based on co-spray-dried powders. Powder Technol..

[4] Singh M.N., Hemant K.S.Y., Ram M., Shivakumar H.G. (2010). Microencapsulation: A promising technique for controlled drug delivery. Res. Pharm. Sci..

[5] Cestari S.H., Bastos K. J., Di Stasi L.C. (2011). Intestinal anti-inflammatory activity of *Baccharis dracunculifolia* in the trinitrobenzenesulphonic acid model of rat colitis. Evid.-Based Complement. Altern. Med..

[6] Figueiredo-Rinhel A.S.G., Kabeya L.M., Bueno P.C.P., Jorge-Tiossi R.F., Azzolini A.E.C.S., Bastos J.K., Lucisano-Valim Y.M. (2013). Inhibition of the human neutrophil oxidative metabolism by *Baccharis dracunculifolia* DC (Asteraceae) is influenced by seasonality and the ratio of caffeic acid to other phenolic compounds. J. Ethnopharmacol..

[7] Dos Santos D.A., Fukui M.J., Dhammika N.N.P., Khan S.I., Sousa J.P.B., Bastos J.K., Andrade S.F., Da Silva A.A.F., Quintão N.L.M. (2010). Anti-inflammatory and antinociceptive effects of *Baccharis dracunculifolia* DC (Asteraceae) in different experimental models. J. Ethnopharmacol..

[8] Hocayen P.D.A., Grassiolli S., Leite N.C., Pochapski M.T., Pereira R.A., da Silva L.A., Snack A.L., Michel R.G., Kagimura F.Y., da Cunha M.A. (2016). *Baccharis dracunculifolia* methanol extract enhances glucose-stimulated insulin secretion in pancreatic islets of monosodium glutamate induced-obesity model rats. Pharm. Biol..

[9] Pereira C.A., Costa A.C.B.P., Liporoni P.C.S., Rego M.A., Jorge A.O.C. (2016). Antibacterial activity of *Baccharis dracunculifolia* in planktonic cultures and biofilms of *Streptococcus mutans*. J. Infect. Public Health.

[10] Guimarães N.S.S., Mello J.C., Paiva J.S., Bueno P.C.P., Berretta A.A., Torquato R.J., Nantes I.L., Rodrigues T. (2012). *Baccharis dracunculifolia*, the main source of green propolis, exhibits potent antioxidant activity and prevents oxidative mitochondrial damage. Food Chem. Toxicol..

[11] Kagimura F.Y., da Cunha M.A.A., Barbosa A.M., Dekker R.F.H., Malfatti C.R.M. (2015). Biological activities of derivatized d-glucans: A review. Int. J. Biol. Macromol..

[12] Magnani M., Castro-Gómez R.J.H. (2008). β-glucana de *Saccharomyces cerevisiae*: Constituição, bioatividade e obtenção. Semin. Ciências Agrárias.

[13] Vetvicka V. (2011). Glucan-immunostimulant, adjuvant, potential drug. World J. Clin. Oncol..

[14] Ajazuddin, Saraf S. (2010). Applications of novel drug delivery system for herbal formulations. Fitoterapia.

[15] Ye Q., Woo M.W., Selomulya C. (2019). Modification of molecular conformation of spray-dried whey protein microparticles improving digestibility and release characteristics. Food Chem..

[16] Walz M., Hirth T., Weber A. (2018). Investigation of chemically modified inulin as encapsulation material for pharmaceutical substances by spray-drying. Colloids Surf. A Physicochem. Eng. Asp..

[17] Li X., Anton N., Arpagaus C., Belleteix F., Vandamme T.F. (2010). Nanoparticles by spray drying using innovative new technology: The Büchi Nano Spray Dryer B-90. J. Control. Release.

[18] Schuck P., Dolivet A., Méjean S., Zhu P., Blanchard E., Jeantet R. (2009). Drying by desorption: A tool to determine spray drying parameters. J. Food Eng..

[19] Haque M.A., Timilsena Y.P., Adhikari B. (2015). Book chapter Spray drying. Drying Technologies or Foods: Fundamentals & Applications.

[20] Bakry A.M., Abbas S., Ali B., Majeed H., Abouelwafa M.Y., Mousa A., Liang L. (2016). Microencapsulation of oils: A comprehensive review of benefits, techniques, and applications. Compr. Rev. Food Sci. Food Saf..

[21] Galanakis C.M., Kotisiou K. (2016). Recovery of bioactive compounds from olive mill waste. Olive mill Waste: Recent Advances for Sustainable Management.

[22] Roleira F.M.F., Tavares-da-Silva E.J., Varela C.L., Costa S.C., Silva T., Garrido J., Borges F. (2015). Plant derived and dietary phenolic antioxidants: Anticancer properties. Food Chem..

[23] da Silva Carvalho A.G., da Costa Machado M.T., da Silva V.M., Sartoratto A., Rodrigues R.A.F., Hubinger M.D. (2016). Physical properties and morphology of spray dried microparticles containing anthocyanins of jussara (*Euterpe edulis Martius*) extract. Powder Technol..

[24] Krishnaiah D., Sarbatly R., Nithyanandam R. (2012). Microencapsulation of *Morinda citrifolia* L. extract by spray-drying. Chem. Eng. Res. Des..

[25] Danaei M., Dehghankhold M., Ataei S., Hasanzadeh Davarani F., Javanmard R., Dokhani A., Khorasani S., Mozafari M. (2018). Impact of particle size and polydispersity index on the clinical applications of lipidic nanocarrier systems. Pharmaceutics.

[26] Singh R., Lillard J.W. (2009). Nanoparticle-based targeted drug delivery. Exp. Mol. Pathol..

[27] Gomes J.F.P.S., Rocha S., Pereira M.D.C., Peres I., Moreno S., Toca-Herrera J., Coelho M.A.N. (2010). Lipid/particle assemblies based on maltodextrin–gum arabic core as bio-carriers. Colloids Surf. B Biointerfaces.

[28] Nawrocka A., Szymańska-Chargot M., Miś A., Wilczewska A.Z., Markiewicz K.H. (2017). Effect of dietary fibre polysaccharides on structure and thermal properties of gluten proteins—A study on gluten dough with application of FT-Raman spectroscopy, TGA and DSC. Food Hydrocoll..

[29] Meng Q., Li Y., Xiao T., Zhang L., Xu D. (2017). Antioxidant and antibacterial activities of polysaccharides isolated and purified from *Diaphragma juglandis* fructus. Int. J. Biol. Macromol..

[30] Iurckevicz G., Marques P.T., Lima V.A. (2017). Análise Química e Quimiométrica de Matrizes de Amido Modificado por Trimetafosfato de Sódio. Rev. Virtual Quim.

[31] Kagimura F.Y., da Cunha M.A.A., Theis T.V., Malfatti C.R.M., Dekker R.F.H., Barbosa A.M., Teixeira S.D., Salomé K. (2015). Carboxymethylation of (1→6)-β-glucan (lasiodiplodan): Preparation, characterization and antioxidant evaluation. Carbohydr. Polym..

[32] Ballesteros L.F., Ramirez M.J., Orrego C.E., Teixeira J.A., Mussatto S.I. (2017). Encapsulation of antioxidant phenolic compounds extracted from spent coffee grounds by freeze-drying and spray-drying using different coating materials. Food Chem..

[33] Surikutchi B.T., Patil S.P., Shete G., Patel S., Bansal A.K. (2013). Drug-excipient behavior in polymeric amorphous solid dispersions. J. Excipients Food Chem..

[34] Horst M.F., Coral D.F., van Raap M.B.F., Alvarez M., Lassalle V. (2017). Hybrid nanomaterials based on gum arabic and magnetite for hyperthermia treatments. Mater. Sci. Eng. C.

[35] Ali A., Ganie S.A., Mazumdar N. (2018). A new study of iodine complexes of oxidized gum arabic: An interaction between iodine monochloride and aldehyde groups. Carbohydr. Polym..

[36] Gómez-Ordóñez E., Rupérez P. (2011). FTIR-ATR spectroscopy as a tool for polysaccharide identification in edible brown and red seaweeds. Food Hydrocoll..

[37] Alipour H.J., Rezaei M., Shabanpour B., Tabarsa M. (2018). Effects of sulfated polysaccharides from green alga Ulva intestinalis on physicochemical properties and microstructure of silver carp surimi. Food Hydrocoll..

[38] Quintanilha R.C., Orth E.S., Grein-Iankovski A., Riegel-Vidotti I.C., Vidotti M. (2014). The use of gum arabic as “Green” stabilizer of poly(aniline) nanocomposites: A comprehensive study of spectroscopic, morphological and electrochemical properties. J. Colloid Interface Sci..

[39] Anjos O., Campos M.G., Ruiz P.C., Antunes P. (2015). Application of FTIR-ATR spectroscopy to the quantification of sugar in honey. Food Chem..

[40] Xu J., Liu W., Yao W., Pang X., Yin D., Gao X. (2009). Carboxymethylation of a polysaccharide extracted from *Ganoderma lucidum* enhances its antioxidant activities in vitro. Carbohydr. Polym..

[41] Wang J., Zhang L. (2009). Structure and chain conformation of five water-soluble derivatives of a β-Dglucan isolated from *Ganoderma lucidum*. Carbohydr. Res..

[42] Castro-Cabado M., Casado A.L., San Román J. (2016). Bio-based thermosets: Effect of the structure of polycarboxylic acids on the thermal crosslinking of maltodextrins. Eur. Polym. J..

[43] Smrčková P., Horský J., Šárka E., Koláček J., Netopilík M., Walterová Z., Kruliš Z., Synytsya A., Hrušková K. (2013). Hydrolysis of wheat B-starch and characterisation of acetylated maltodextrin. Carbohydr. Polym..

[44] Silva D.A., Costa D.A., Silva D.F., Souza M.F.V., Agra M.F., Medeiros I.A., Barbosa-Filho J.M., Braz-Filho R. (2005). Flavonóides glicosilados de *Herissantia tiubae* (K. Schum) Brizicky (Malvaceae) e testes farmacológicos preliminares do canferol 3,7-di-O-α-L-ramnopiranosídeo. Braz. J. Pharmacogn..

[45] Santos A.M.P., Bertoli A.C., Carolina A., Borges C.P., Gomes R.A.B., Garcia J.S., Trevisan M.G. (2018). New organomineral complex from humic substances extracted from poultry wastes: synthesis, characterization and controlled release study. J. Braz. Chem. Soc..

[46] Lima I.A.D., Khalil N.M., Tominaga T.T., Lechanteur A., Sarmento B., Mainardes R.M. (2018). Mucoadhesive chitosan-coated PLGA nanoparticles for oral delivery of ferulic acid. Artif. Cells Nanomed. Biotechnol..

[47] Su S.-F., Chou C.-H., Kung C.-F., Huang J. (2003). In vitro and in vivo comparison of two diclofenac sodium sustained release oral formulations. Int. J. Pharm..

[48] Gibaldi M., Feldman S. (1967). Establishment of sink conditions in dissolution rate determinations. Theoretical considerations and application to nondisintegrating dosage forms. J. Pharm. Sci..

[49] Korsmeyer R.W., Gurny R., Doelker E., Buri P., Peppas N.A. (1983). Mechanisms of solute release from porous hydrophilic polymers. Int. J. Pharm..

[50] Siepmann J., Peppas N.A. (2011). Higuchi equation: Derivation, applications, use and misuse. Int. J. Pharm..

[51] Herculano E.D., de Paula H.C.B., de Figueiredo E.A.T., Dias F.G.B., Pereira V.D.A. (2015). Physicochemical and antimicrobial properties of nanoencapsulated *Eucalyptus staigeriana* essential oil. LWT Food Sci. Technol..

[52] Agrawal A., Purwar R. (2018). Swelling and drug release kinetics of composite wound dressing. Indian J. Fibre Text. Res..

[53] Fu Y., Kao W.J. (2010). Drug release kinetics and transport mechanisms of non-degradable and degradable polymeric delivery systems. Expert Opin. Drug Deliv..

[54] Rigter P.L., Peppas N.A. (1987). A simple equation for description of solute relase I. Fickian and non-fickian release from non-swellable devices in the form of slabs, spherer, cylinders or discs. J. Control. Release.

[55] Romero A.I., Villegas M., Cid A.G., Parentis M.L., Gonzo E.E., Bermúdez J.M. (2018). Validation of kinetic modeling of progesterone release from polymeric membranes. Asian J. Pharm. Sci..

[56] Siepmann J., Peppas N. (2001). Modeling of drug release from delivery systems based on hydroxypropyl methylcellulose (HPMC). Adv. Drug Deliv. Rev..

[57] Prajapati S.K., Richhaiya R., Singh V.K., Singh A.K., Kumar S., Chaudhary R.K. (2012). Formulation and evaluation of once daily sustained release matrix tablets of aceclofenac using natural gums. J. Drug Deliv. Ther..

[58] Sitta D.L.A., Guilherme M.R., da Silva E.P., Valente A.J.M., Muniz E.C., Rubira A.F. (2014). Drug release mechanisms of chemically cross-linked albumin microparticles: Effect of the matrix erosion. Colloids Surf. B Biointerfaces.

[59] Bruschi M.L. (2015). 5—Mathematical models of drug release. Strategies to Modify the Drug Release from Pharmaceutical Systems.

[60] Marques G.R., Borges S.V., de Mendonça K.S., de Barros Fernandes R.V., Menezes E.G.T. (2014). Application of maltodextrin in green corn extract powder production. Powder Technol..

[61] de Barros Fernandes R.V., Borges S.V., Botrel D.A. (2014). Gum arabic/starch/maltodextrin/inulin as wall materials on the microencapsulation of *rosemary* essential oil. Carbohydr. Polym..

[62] Teixeira M.I., Andrade L.R., Farina M., Rocha-Leão M.H.M. (2004). Characterization of short chain fatty acid microcapsules produced by spray drying. Mater. Sci. Eng. C.

[63] Rezende T., Corrêa J., Aarestrup B., Aarestrup F., de Sousa O., da Silva Filho A. (2014). Protective effects of *Baccharis dracunculifolia* leaves extract against carbon tetrachloride- and acetaminophen-induced hepatotoxicity in experimental animals. Molecules.

[64] de Sousa J.P.B., da Silva Filho A.A., Bueno P.C.P., Gregório L.E., Furtado N.A.J.C., Jorge R.F., Bastos J.K. (2009). A validated reverse-phase HPLC analytical method for the quantification of phenolic compounds in *Baccharis dracunculifolia*. Phytochem. Anal..

[65] Dias M.P., Nozari R.M., Santarém E.R. (2017). Herbicidal activity of natural compounds from *Baccharis* spp. on the germination and seedlings growth of *Lactuca sativa* and *Bidens pilosa*. Allelopath. J..

[66] Cunha M.A.A., Turmina J.A., Ivanov R.C., Barroso R.R., Marques P.T., Fonseca E.A.I., Fortes Z.B., Dekker R.F.H., Khaper N., Barbosa A.M. (2012). Lasiodiplodan, an exocellular (1→6)-β-d-glucan from Lasiodiplodia theobromae MMPI: production on glucose, fermentation kinetics, rheology and anti-proliferative activity. J. Ind. Microbiol. Biotechnol..

[67] Salgado M., Rodríguez-Rojo S., Alves-Santos F.M., Cocero M.J. (2015). Encapsulation of resveratrol on lecithin and β-glucans to enhance its action against *Botrytis cinerea*. J. Food Eng..

[68] Aburto L.C., Tavares D.D.Q., Martucci E.T. (1998). Microencapsulação de óleo essencial de laranja. Food Sci. Technol..

[69] Saikia S., Kumar Mahnot N., Lata Mahanta C. (2015). Optimisation of phenolic extraction from *Averrhoa carambola* pomace by response surface methodology and its microencapsulation by spray and freeze drying. Food Chem..

[70] Singleton V.L., Rossi J.A. (1965). Colorimetry of total phenolics with phosphomolybdic-phosphotungstic acid reagents. Am. J. Enol. Vitic..

[71] Brand-Williams W., Cuvelier M.E., Berset C. (1995). Use of a free radical method to evaluate antioxidant activity. LWT Food Sci. Technol..

[72] Huang M.-H., Huang S.-S., Wang B.-S., Wu C.-H., Sheu M.-J., Hou W.-C., Lin S.-S., Huang G.-J. (2010). Antioxidant and anti-inflammatory properties of *Cardiospermum halicacabum* and its reference compounds ex vivo and in vivo. J. Ethnopharmacol..

[73] Wootton-Beard P.C., Ryan L. (2011). A beetroot juice shot is a significant and convenient source of bioaccessible antioxidants. J. Funct. Foods..

[74] Rosa M.F., Vilhena R.D.O. (2012). Dissolução intrínseca: conceito e aplicações na indústria farmacêutica. Rev. Eletrônica Farmácia.

